# Presence and Characterisation of Anaemia in Diabetic Foot Ulceration

**DOI:** 10.1155/2014/104214

**Published:** 2014-07-23

**Authors:** J. A. Wright, M. J. Oddy, T. Richards

**Affiliations:** ^1^Department of Vascular Surgery, Royal Free and University College Hospitals, London, UK; ^2^Department of Orthopaedic Surgery, University College Hospital, London, UK

## Abstract

*Introduction*. Diabetic foot ulceration (DFU) is the commonest cause of severe limb ischaemia in the western world. In diabetes mellitus, anaemia is frequently unrecognized, yet studies have shown that it is twice as common in diabetics compared with nondiabetics. We aimed to assess the incidence of anaemia and further classify the iron deficiency seen in a high-risk DFU patient group. *Methods*. An observational study was undertaken in a multidisciplinary diabetic foot clinic setting. All patients with DFU attending over a four-month period were included. Anaemia was defined as haemoglobin (Hb) levels <12 g/dL. Iron deficiency was classified according to definitions of AID (absolute iron deficiency) and FID (functional iron deficiency). *Results*. 27 patients had DFU; 14 (51.9%) were anaemic; two (7.41%) had severe anaemia (Hb < 10 g/dL). No patient had B12 or Folate deficiency. In patients with anaemia, there was significant spread of indices. Only one patient had “textbook” absolute iron deficiency (AID) defined as low Hb, MCV, MCH, and ferritin. Functional iron deficiency (FID) was seen in a further seven patients (25.5%). *Conclusion*. Anaemia and iron deficiency are a common problem in patients with DFU. With current clinical markers, it is incredibly difficult to determine causal relationships and further in-depth scientific study is required.

## 1. Introduction

Since the earliest descriptions by Aretaeus of Cappadocia in the 2nd century AD, “*Diabetes is a dreadful affliction, not very frequent among men, being a melting down of the flesh and limgs [sic] into urine…life is short, unpleasant and painful*” [1]; diabetes mellitus remains one of the most serious worldwide health challenges.

Diabetic foot ulceration (DFU) continues to be the commonest cause of severe limb ischaemia in vascular surgery. Up to 25% of diabetic patients are at risk of developing DFU during their lifetime and poor wound healing is a principle reason for morbidity and mortality [2]. Diabetes carries an increased risk of a person undergoing lower extremity amputation over twenty times that of age-matched healthy individuals [3].

The pathophysiology of DFU is complex and the reasons for slow and poor healing are incompletely understood. It is known that micro- and macrovascular disease, dysfunctional glycaemic control, polyneuropathy, foot deformity, altered biomechanics, active infection, inflammation and impaired immunity are of key importance and associated with poor outcome [4]. Clinically, crucial aspects of therapy to promote wound healing include prompt revascularisation, offloading, and treatment of infection [5].

As with many chronic diseases, anaemia is found in diabetes but is frequently unrecognized. Studies suggest that anaemia is twice as common in diabetics compared with nondiabetics [6–8]. Development of anaemia is an additional burden to the microvascular complications of diabetes [9]. To date the association between anaemia and DFU has undergone limited study as most literature investigating diabetes and anaemia has been led by nephrologists and diabetes is the leading cause of “renal anaemia” [10].

The overall adult anaemia “cutoffs” or definitions have been unchanged since 1968. By the time anaemia is detected, iron deficiency is usually advanced. Iron deficiency has consequences even when no anaemia is apparent clinically [11].

Traditionally, anaemic patients have been divided into two groups: those with “iron deficiency anaemia” (IDA) and those with “anaemia of chronic disease” (ACD). IDA is classically defined as microcytic hypochromic anaemia, but ACD can be difficult to diagnose, often considered a diagnosis of exclusion. This is because chronic disease and inflammation are associated with elevated ferritin as part of the acute-phase response. The alternative terminology “anaemia of inflammation” is also in use.

**Figure 1:**
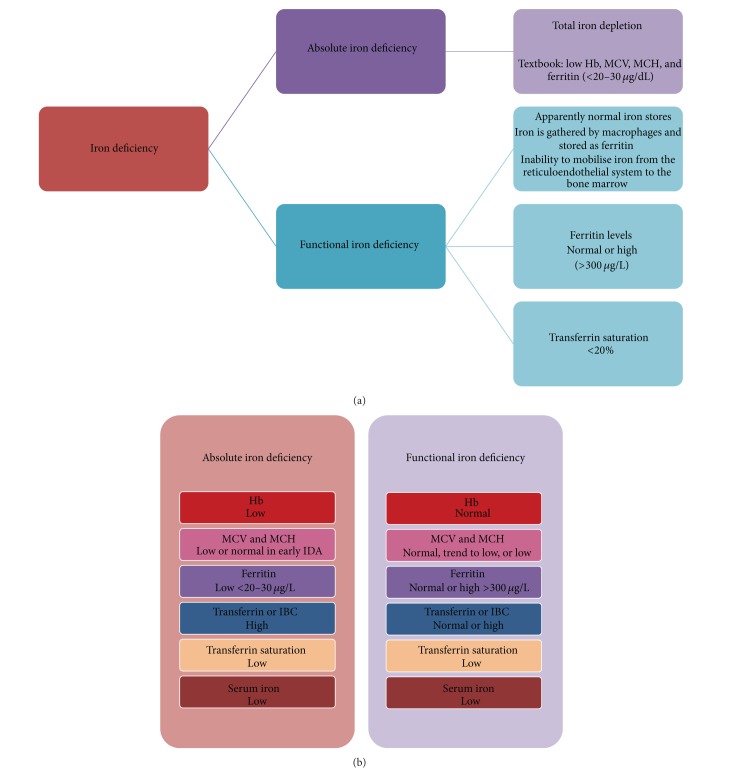
(a) Definitions of iron deficiency. (b) Interpretation of blood investigations in absolute iron deficiency and functional iron deficiency.

More recently, definitions of “absolute” (AID) and “functional iron deficiency” (FID) have been proposed [12]; see Figures 1(a) and 1(b). FID is characterised by an inflammatory state where iron is gathered by macrophages and stored as ferritin with failure of delivery of iron from the reticuloendothelial system to the bone marrow. Although iron stores appear normal, the pathways for delivery to the place of need (bone marrow) are blocked.

The associations between anaemia and DFU are poorly understood. A previous retrospective study by our group has suggested correlation between anaemia and clinical stage of DFU [13]. To date, there have been no studies that have characterized the cause of anaemia seen in DFU.

## 2. Aims

Our specific aims were to assess the incidence of anaemia in patients presenting with severe (high-risk) DFU. Secondly, in order to optimize diabetic foot-care and develop therapeutic strategies, we wished to characterize the anaemia seen into AID and FID.

## 3. Research Design and Methods

A prospective observational study of a consecutive cohort of DFU patients receiving outpatient multidisciplinary diabetic-foot care at a university teaching hospital was undertaken. All patients with severe DFU (Texas classification IIc&d/IIIc&d or Wagner grades 2–5) attending over a four-month period (1 September 2010 to 31 December 2010) were included. As part of their routine medical care, these patients attended initial and 3-month follow-up outpatient diabetic-foot care appointments.

At the initial outpatient diabetic-foot care visit, in all severe DFU patients we assessed the following investigations: blood-tests including haemoglobin (Hb), B12, Folate, iron studies, reticulocyte count, C-reactive protein (CRP), erythrocyte sedimentation rate (ESR), full blood count, renal profile, and glycated haemoglobin (HbA1c). These investigations were repeated at three-month follow-up assessment. Additionally, we assessed outcome of wound-healing status (healed or nonhealed) at three months.

Anaemia was defined as haemoglobin (Hb) levels <12 g/dL; severe anaemia was defined as Hb <10 g/dL. Classification of DFU patients anaemia at both initial and three-month follow-up assessment was undertaken. Iron deficiency was classified according to definitions of AID (total body iron depletion) and FID (apparently normal iron stores with inability to mobilise iron from reticuloendothelial system), with blood investigations assessed according to Figure 1(b) and our hospital laboratory normal reference ranges.

At our centre, multidisciplinary diabetic foot care is established with overarching clinical governance and care pathways under vascular surgery. Patient referrals were received directly from three principal primary care trusts with some tertiary referrals nationally. In this study, no patient included underwent diabetic foot care at institutions elsewhere. Those patients taking ferrous sulphate or vitamin supplements continued to do so through the study. Patients with other foot deformities, Charcot foot, gout, and ischaemic or venous ulceration were excluded. Patients requiring renal replacement therapy at the time of study were also excluded as this service was not provided on-site. Approval for this study was granted by our hospital clinical audit department.

Statistical analysis was performed using Prism for Windows version 6.0 (GraphPad Software, La Jolla, CA, USA; http://www.graphpad.com/). Continuous data are expressed as median ± range or mean ± standard deviation as appropriate. For comparisons of both initial and three-month nonanaemic and anaemic patients investigations, Mann-Whitney *t*-tests (for white blood cell count, creatinine, CRP, and ESR) and unpaired *t*-tests with welsh correction (for eGFR) were applied. All data were subject to normality testing and two-tailed *P* values were quoted. Spearman rank correlation was performed to assess presence of relationship between patients initial and three-month follow-up CRP and Ferritin levels, with two-tailed *P* and *R* values quoted. To assess associations between anaemia and wound healing and endovascular intervention, Fisher's exact test was performed, with *P* value quoted.

## 4. Results

### 4.1. Demographics

Of the 218 vascular outpatient cases seen, 27 patients with severe DFU (Texas classification IIc&d/IIIc&d or Wagner grades 2–5) were identified over a four-month period. Of these patients, 22 (81.5%) were male; median age was 67 years (range 27–86). Median initial HbA1c of all patients was 62.8 mmol/mol (<30–121.9 mmol/mol). Only 3 patients (16.6%) continued ferrous sulphate treatment throughout the study. No patient underwent blood transfusion within the study period. At three-month followup, a further five patients did not undergo further investigations to determine presence or characterization of anaemia; in one case this was due to hospital admission at another institution, one was due to transfer of outpatient care to another institution, and three patients were lost to followup. No patient died during the study period.

### 4.2. Initial Presence and Characterisation of Anaemia

**Figure 2:**
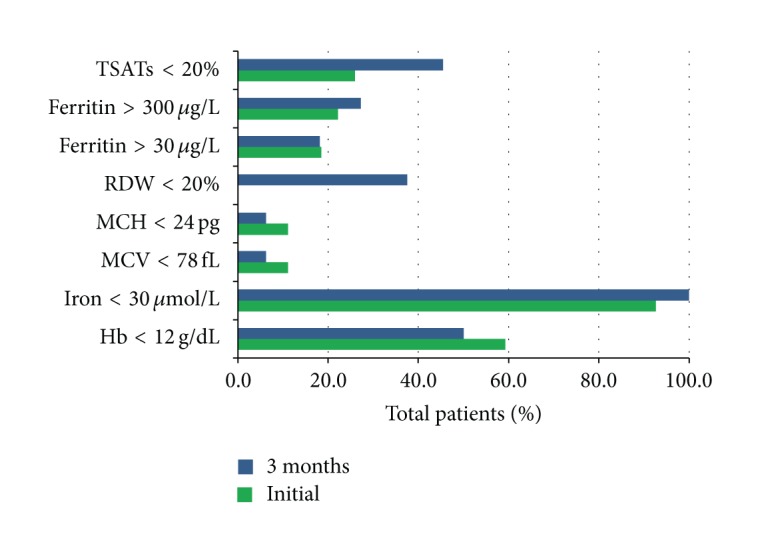
Percentage of initial and three-month follow-up patients with abnormal indices and iron studies.* Definitions of abnormal indices listed are indicated on the y-axis. Key: percentage of initial follow-up patients with abnormal indices is indicated in green; three-month follow-up patients are indicated in blue*.

Half, 14 patients (51.9%), were anaemic (Hb < 12 g/dL) at initial presentation. Two patients (7.4%) had severe anaemia (Hb < 10 g/dL). The median initial Hb of all patients was 11.60 g/dL (range 7.90–16.50 g/dL). Median initial HbA1c of anaemic patients was 67.2 mmol*·*mol (44.3–102.2 mmol/mol). No patient had B12 or Folate deficiency. In patients with anaemia, there was significant spread of iron studies (see Figure 2 and Table 1). Only one patient had AID. FID was seen in seven “nonanaemic” patients.* Thus for definitions of anaemia and iron deficiency combined, twenty-one (77.8%) severe DFU patients could be classified in this abnormal group.*

### 4.3. Persistence of Anaemia at Three-Month Followup

At three-month followup, half (50.0%) of the patients were anaemic. In all patients who were initially anaemic (by definition: Hb < 12 g/dL), their anaemia persisted and did not resolve. The median follow-up Hb was 11.65 g/dL (range 10.0–14.1 g/dL). The difference between patients initial and three-month Hb decreased over the study period; mean decrease was 0.21 g/dL (SD 1.34). None of the nonanaemic patients became anaemic over the three-month period.

### 4.4. Haematological, Renal, and Inflammatory Markers

There was no significant difference between anaemic and nonanaemic patients' initial white blood cell count, creatinine, eGFR, and C-reactive protein. In anaemic patients, there was a significantly elevated initial ESR (*P* = 0.0065); see Table 2. There was no significant difference between anaemic and nonanaemic white blood cell count, creatinine, eGFR, C-reactive protein, and ESR.

There was no significant correlation between patients CRP or Ferritin levels at initial presentation (*two-tailed P* = 0.2149*, Spearman rank R* = 0.2466) or at three months (*two-tailed P* = 0.5307,* Spearman rank R* = 0.2270).

### 4.5. Relationship between Anaemia, Wound Healing, and Endovascular Intervention

Five patients had healed during the three-month follow-up period. Initial presence or absence of anaemia (Hb < 12 g/dL) was not associated with healing status at three months (two-tailed *P* = 0.3705, Fisher's exact test).

Interestingly, five separate patients required admission for endovascular interventions (including crural angioplasty or stenting), all of whom were anaemic with Hb <12 g/dL (two of these had severe anaemia Hb < 10 g/dL). This association between anaemia and endovascular intervention approached statistical significance, *P* = 0.0598, Fisher's exact test.

## 5. Discussion

This prospective study showed a high incidence of anaemia in patients with severe DFU. It demonstrates the significant difficulties encountered in the interpretation of iron indices to provide clear classification of anaemia seen in severe DFU.

We found that at initial assessment, sixteen patients (59.3%) were classified as anaemic by definition Hb < 12 g/dL. Only one of these patients had absolute or textbook IDA. This finding is comparable to earlier studies undertaken in developing countries where percentages of DFU patients with IDA range from 49% to 62%. In a cross-sectional study of fifty DFU patients attending a Nigerian hospital, 49% of patients had IDA at the time of their presentation [14]. In a similar prospective case series of forty-seven DFU patients (this patient cohort included Wagner grades 2-3) 57% of patients were classified as “anaemic” [15]. Further cross sectional study of forty-two Nigerian patients with all grades of DFU (Wagner grade 1–5) showed that 61.8% of patients had “anaemia,” which was found to be a significant risk factor for in-hospital mortality [16]. Our study in the UK provides some evidence to suggest that the anaemia observed in severe DFU patients may be more than nutritional in its nature.

**Figure 3:**
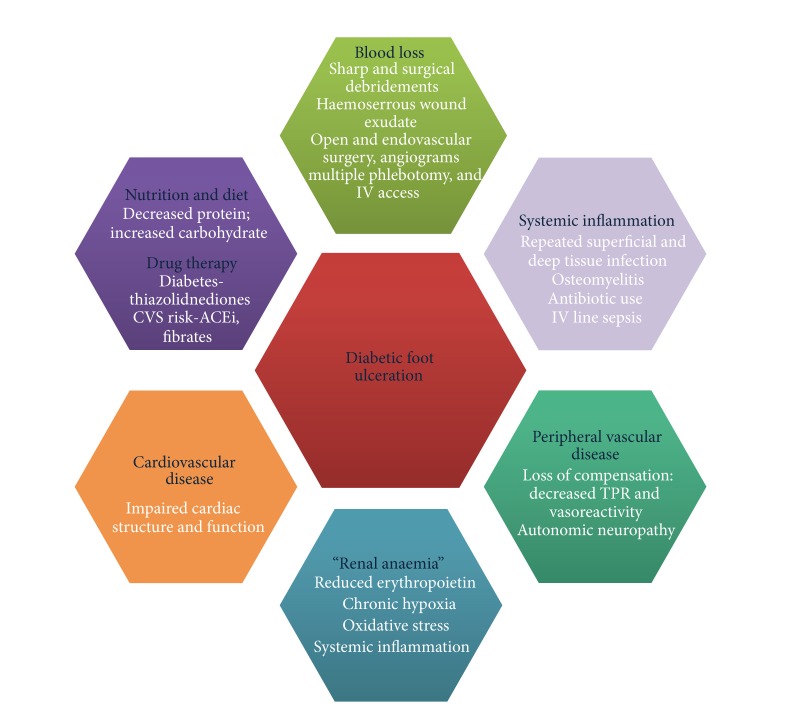
Mechanisms contributing to anaemia in poorly healing diabetic foot ulceration.

Additionally, we found that for definitions of anaemia and iron deficiency combined, twenty-one (77.8%) severe DFU patients were classified as abnormal. There was a high initial incidence of altered haematinics and FID. Although the handful of studies investigating anaemia in DFU mentioned above document presence of anaemia, they do not include full assessment of iron indices or attempt classification of DFU patients into those with FID. In a study of UK patients with diabetes, the “Teesside Anaemia in Diabetes Study” found a prevalence of altered haematinics of 40% [17]. We suggest that there are multiple factors contributing to the presence of anaemia in DFU; see Figure 3.

Studies of anaemic diabetic patients have found that they have a higher rate of stroke, ischaemic heart disease, hypertension, and CKD, as mentioned above [18]. In an analysis of seven UK based diabetic patient cohorts, Kengne et al. [19] found that anaemia and CVD conferred similar mortality risks. Studies of patient outcomes in anaemic DFU patients are limited. In a single study of one hundred and eighty DFU patients, anaemia was associated with adverse wound-healing outcomes [20]. In our study, we did not detect any significant association between anaemia and major or minor amputation or mortality. This is likely due to the low numbers of patients included and positive outcomes experienced at our centre which has a proactive multidisciplinary approach to diabetic foot care. Currently, retrospective analysis of existing hospital-based and research databases is virtually impossible owing to lack of haematinic investigations being undertaken and recorded in this severe DFU patient group and hospital coding systems which do not permit identification of those patients suffering from diabetic foot disease.

The impact of anaemia on cardiovascular function in DFU patients has undergone limited investigation. The ACORD trial (Anaemia CORrection in Diabetes) investigated the effects of anaemia correction with epoetin beta on cardiac structure and function in patients with early diabetic nephropathy and moderate LVH. Normalisation of Hb level (to target 13–15 g/dL) did not decrease LVMI but did prevent additional increase in LVH and improved quality of life [21]. Further studies in diabetic patients undertaken by Srivastava et al. [22] found that anaemia was associated with cardiac dysfunction and a correlation with plasma markers of cardiac risk, including BNP, CRP, and AVP. Whilst these studies suggest that diabetes-related anaemia is associated with cardiovascular dysfunction, only one study has addressed such cardiovascular dysfunction specifically in a DFU patient cohort [23]. Furthermore, there are no studies that have investigated the effect of iron correction treatment (erythropoietin or IV iron) on DFU healing rates.

Patients with severe DFU require multiple endovascular intervention. We found that all severe DFU patients requiring endovascular intervention were classified as anaemic. This finding is consistent with recent literature that has shown that, in diabetic patients with PVD, Hb decline correlates with both clinical symptom deterioration and disease progression [24]. Furthermore, in diabetic patients undergoing open-bypass surgery for PVD, preoperative low Hb is associated with major cardiac events and death [25]. Further study is required to establish causal relationships. The impact of anaemia on both the macrovascular and microvascular diseases seen in DFU requires more specific study.

CKD is an independent risk factor for the development of foot lesions in the diabetic population [26]. We found that there was no significant difference between anaemic and nonanaemic patients' initial and three-month follow-up creatinine and eGFR. This is due to the fact that we did not include DFU patients undergoing renal replacement therapy, as this service is provided at a different hospital site. More recent studies have suggested that screening for anaemia in current diabetes management should be extended. New et al. [27] found that, below an eGFR of 83 mL/min/1.73 m^2^, for every 1 mL/min/1.73 m^2^ fall in eGFR, there was an associated 0.4 (0.3–0.5) g/L fall in haemoglobin. The contribution of “renal-anaemia” to poor wound healing seen in DFU requires further investigation.

We also found that anaemic patients had a significantly elevated initial ESR, although no significant difference was found between anaemic and nonanaemic patients' initial and three-month follow-up white blood cell count and C-reactive protein. In the study by Ekpebegh et al. [16], leucocytosis was shown to be a significant risk factor for inpatient mortality, although association between Hb decline and other inflammatory markers was not described. Our finding of ESR being elevated at initial visit is most likely related to the inflammatory and infective processes occurring in DFU. Although further experimental study of hepcidin levels would be of interest, in order to fully characterize the anaemia seen in severe DFU. Hepcidin binds to ferroportin (iron export protein) present on macrophages leading to iron trapping and further FID [28].

The relationships between anaemia and wound-healing outcomes (wound-healing rates, healing following amputation) are difficult to comment on based on the findings of this study. Only five patients had healed during the three-month follow-up period and presence or absence of anaemia was not associated with healing status at three months. Furthermore, the impact of AID and FID on wound healing could not be determined owing to the current significant difficulties encountered in the interpretation of iron indices; the underlying pathophysiological mechanisms require detailed scientific study.

## 6. Conclusion

Anaemia and iron deficiency are a common problem in patients with DFU. Identification of both the presence of anaemia and FID in DFU patients is necessary to assess the role of iron replacement and therapeutic strategies. With current clinical markers, it is incredibly difficult to determine causal relationships. In-depth scientific study of the mechanisms underlying poorly healing DFU in the presence of AID and FID is required.

## Figures and Tables

**Table 1:** Initial DFU patients indices and iron studies: functional and absolute iron deficiency.

Indices and iron studies	Nonanaemic patients with functional iron deficiency		Anaemic patients with absolute iron deficiency∗		Anaemic patients with abnormal indices and iron studies∗∗	
	Median values	Range	Value	Range	Median values	Range
Haemoglobin g/dL	12.2	12.0–16.5	11.2	—	11.1	7. 0–11.5
MCV fL	91.6	87.1–95.2	72.4	—	93.0	61.6–122.5
MCH pg	29.3	27.0–32.9	21.1	—	29.6	18.6–37.8
RDW %	13.7	12.3–14.5	15.9	—	14.6	13.6–18.7
Ferritin ug/L	131.0	70.0–983.0	13.0	—	125.0	24.0–744.0
Transferrin saturation %	24.0	22.0–49.0	15.1	—	19.0	13.0–45.0
Iron umol/L	12.5	9.0–24.0	8.0	—	12.0	5.0–45.0
Reticulocytes %	1.6	0.5–1.5	0.6	—	1.20	0.6–10.0

Anaemia defined as Hb < 12.0 g/dL. Initial visit patient values presented only. ∗Only one anaemic patient initially had a text book absolute iron deficiency; hence these data are presented without range. ∗∗The remaining thirteen anaemic patients had abnormal iron indices and studies (iron < 30 umol/L, MCH < 24 pg, MCV < 78 fL, RDW < 12%, TSATS < 20%, ferritin < 30 ug/L or >300 ug/L). Normal hospital laboratory reference ranges: MCV fL 80–99; MCH 27.0–33.5; RDW 11.5–15.0; ferritin 300–400 ug/L; transferrin saturation 20–50%; iron 10.6–28.3 umol/L; reticulocytes 0.38–2.64%.

**Table 2:** Nonanaemic and anaemic patients initial and three-month follow-up haematological, renal, and inflammatory markers.

Markers	Initial					Three-month followup				
	Nonanaemic patients		Anaemic patients		Two-tailed *P* value	Nonanaemic patients		Anaemic patients		Two-tailed *P* Value
	Median values	Range	Median values	Range		Median values	Range	Median values	Range	
White blood cell count ×10^9^/L	8.19	6.28–12.78	7.85	4.74–17.05	ns 0.6448	8.70	7.67–18.42	6.87	5.51–12.78	ns 0.2743

Creatinine umol/L	83.0	61.0–234.0	94.0	46.0–364.0	ns 0.6041	86.5	70.0–288.0	109.0	48.0–472.0	ns 0.7618

eGFR mL/min/1.73 sqm	84.0	57.0–90.0	64.5	15.0–90.0	ns 0.1572	78.5	54.0–90.0	59.0	12.0–90.0	ns 0.3536

C-reactive protein (CRP) mg/L	1.70	1.2–49.0	4.50	0.6–84.6	ns 0.4734	8.65	0.6–144.0	3.70	0.6–59.4	ns 0.5596

Erythrocyte sedimentation rate (ESR) mm/hr	5.5	4.0–28.0	14.0	8.0–119.0	∗∗ 0.0065	20.0	16.0–20.0	24.0	13.0–86.0	ns 0.5000

Definition of anaemia: Hb < 12 g/dL. Normal hospital laboratory reference ranges: white blood cell count 3–10 **×** 10^9^/L; creatinine 66–112 umol/L; CRP 0–50 mg/L; ESR 1–20 mm/hr. Mann-Whitney *t*-test applied for comparisons of white blood cell count, creatinine, CRP, and ESR. Unpaired *t*-test with Welsh correction applied for comparisons of eGFR. ∗∗Two-tailed *P* < 0.05.

## Abbreviations

ACD: Anaemia of chronic disease
IDA: Iron deficiency anaemia
AID: Absolute iron deficiency
FID: Functional iron deficiency
eGFR: Estimated glomerular filtration rate
CRP: C-reactive protein
ESR: Erythrocyte sedimentation rate
ACEi: Ace inhibitor
TSATs: Transferrin saturation.
